# Crystal structure of the inclusion complex of cholesterol in β-cyclodextrin and molecular dynamics studies

**DOI:** 10.3762/bjoc.14.69

**Published:** 2018-04-11

**Authors:** Elias Christoforides, Andreas Papaioannou, Kostas Bethanis

**Affiliations:** 1 Department of Biotechnology, Agricultural University of Athens, 75 Iera Odos, Athens 11855, Greece

**Keywords:** beta-cyclodextrin, cholesterol, crystal structure, molecular dynamics

## Abstract

The role of beta-cyclodextrin (β-CD) in cholesterol removal primarily from mammalian cells and secondly from dairy products has been studied thoroughly in recent years. Although the physicochemical characterization of the inclusion compound of cholesterol in β-CD has been achieved by various methods, no crystal structure has been determined so far. We report here the crystal structure of the inclusion compound of cholesterol in β-CD. The inclusion complex crystallizes in the triclinic space group *P*1 forming head-to-head dimers which are stacked along the *c*-axis. One well-defined cholesterol molecule ‘axially’ encapsulated inside the β-CD dimer and 22 water molecules that stabilize the complexes in the crystalline state comprise the asymmetric unit of the structure. The dimers are arranged in an intermediate (IM) channel packing mode in the crystal. Moreover, MD simulations, at 300 and 340 K, based on the crystallographically determined coordinates of the complex show that the formed cholesterol/β-CD inclusion compound remains very stable in aqueous solution at both temperatures.

## Introduction

**Figure 1:**
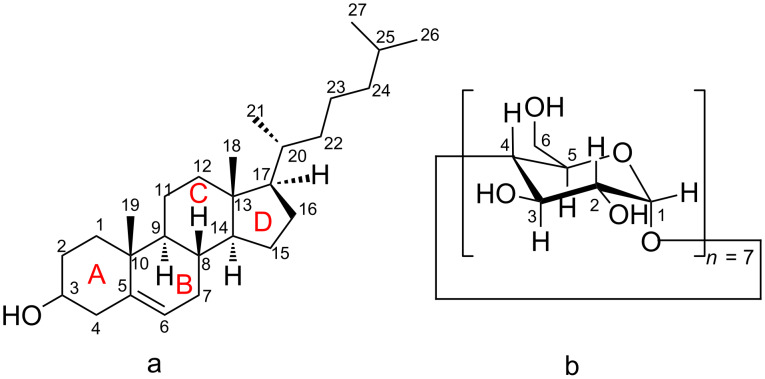
Schematic representation of (a) the cholesterol molecule; (b) the β-cyclodextrin molecule.

Cholesterol ((3β)-cholest-5-en-3-ol, CHL, Figure 1a) is a polycyclic steroid that is synthesized in mammalian cells and has a significant role in biology as an essential structural component of the cell walls and as precursor for the biosynthesis of several substances such as vitamin D, bile acids and steroid hormones. However, the consumption of food rich in cholesterol like meat, eggs and dairy products has been associated with many diseases such as atherosclerosis, hypertension, coronary heart disease, heart stroke and cerebral infarction [1]. Moreover, the abnormal accumulation of cholesterol in endolysosomes emerging from inherited lysosomal storage disorders known as Niemann–Pick type C disease (NPC) leads to various clinical symptoms, such as progressive neurodegeneration and hepatosplenomegaly, often resulting in fatality at an early age [2]. As the cholesterol exchange between tissues at the whole body level and fundamental insights into the physiology of cholesterol trafficking are already known, the development of drug carriers and cell cholesterol removal agents for controlling cholesterol-related disorders [3] are of special interest.

β-Cyclodextrin (β-CD, Figure 1b) is a cyclic polysaccharide consisting of seven α-(1->4)-linked α-D-glucopyranose units and is well known for its ability to form inclusion complexes by entrapping a wide range of guest molecules into its internal hydrophobic cavity. It is non-toxic, non-irritating, edible, chemically stable, easy separable and widely used in pharmaceutical, food and chemical industry [4]. Two major cyclodextrin applications dictate a meticulous study of their inclusion complexes with the cholesterol molecule. β-CD and its modified derivatives (2,6-di-*O*-methyl-β-CD or DM-β-CD, randomly methylated β-CD or RAMEB and 2-hydroxypropyl-β-CD or HP-β-CD) comprise a class of pharmacological agents commonly used to remove membrane cholesterol from cells [5–8]. Cholesterol depletion using CDs is advantageous over the use of binding agents like digitonin, filipin and saponin which are not compatible with live cells. Recently, the use of cyclodextrin as a valuable therapeutic agent for treatment of NPC disease, for which no effective treatment is currently available, has been investigated. It has been shown that administration of HP-β-CD has significantly reduced lysosomal cholesterol accumulation albeit the need for high doses is likely to be detrimental and might cause cell death [9–11]. Sulfobutyl ether-β-CD and sulfobutyl ether-γ-CD showed efﬁcacy with increased safety in NPC animal models [12]. Moreover, superstructures of cyclodextrins like mono-lactose-appended β-CD [13] and biocleavable pluronic/β-CD-based polyrotaxanes [14] as well as PEG-lipid micelles (DSPE-PEG) in combination with HP-β-CD [15] have shown enhanced therapeutic effects and exhibit a reduced toxicity. In food industry β-CD has been used in many applications such as flavor protection and flavor delivery, controlled release of desired constituents and removal and masking of undesirable components [16]. But the most prevalent use of CD in this field is the removal of cholesterol from animal products like milk [17], butter [18], cheese [19] and eggs [20] which contains more than 90% less cholesterol when treated with β-CD. As consumers are becoming more and more concerned about their eating habits, food companies have developed many techniques to reduce cholesterol, as extraction with organic solvents, supercritical carbon dioxide extraction or cholesterol degradation by cholesterol oxidases. But these methods are not selective as other components are also removed and they require expensive equipment and high operational cost [21].

In the past, many studies have been published on the characterization of the cholesterol/β-CD inclusion complex [22], its binding affinity [23–25], the inclusion mode of the complex [26] and its dynamic behavior through MD simulations [27–29] but its crystal structure is absent. In this work, the structure of CHL/β-CD is determined by X-ray crystallography and its geometrical features are examined thoroughly. In order to examine the stability of the crystallographically determined model excluding the crystal contacts observed in the crystalline state, MD simulations of the inclusion complex in aqueous environment were performed. The starting set of coordinates was based on the asymmetric unit of the determined structure and the dynamic behavior of the inclusion complex was monitored at two different temperatures (300 and 340 K) to gain some insight on the evolution of the host–guest interactions and to estimate the host–guest binding affinity in aqueous solution.

An understanding of the structural details of cholesterol inclusion in CDs may be useful in the engineering of modified guest–host preparations with optimized pharmacological properties and shape future therapeutic strategies. Since there are other similar molecules, such as plant sterols that share certain chemical groups, our findings may be relevant for these guests as well.

## Results and Discussion

### Description of the crystal structure

**Figure 2:**
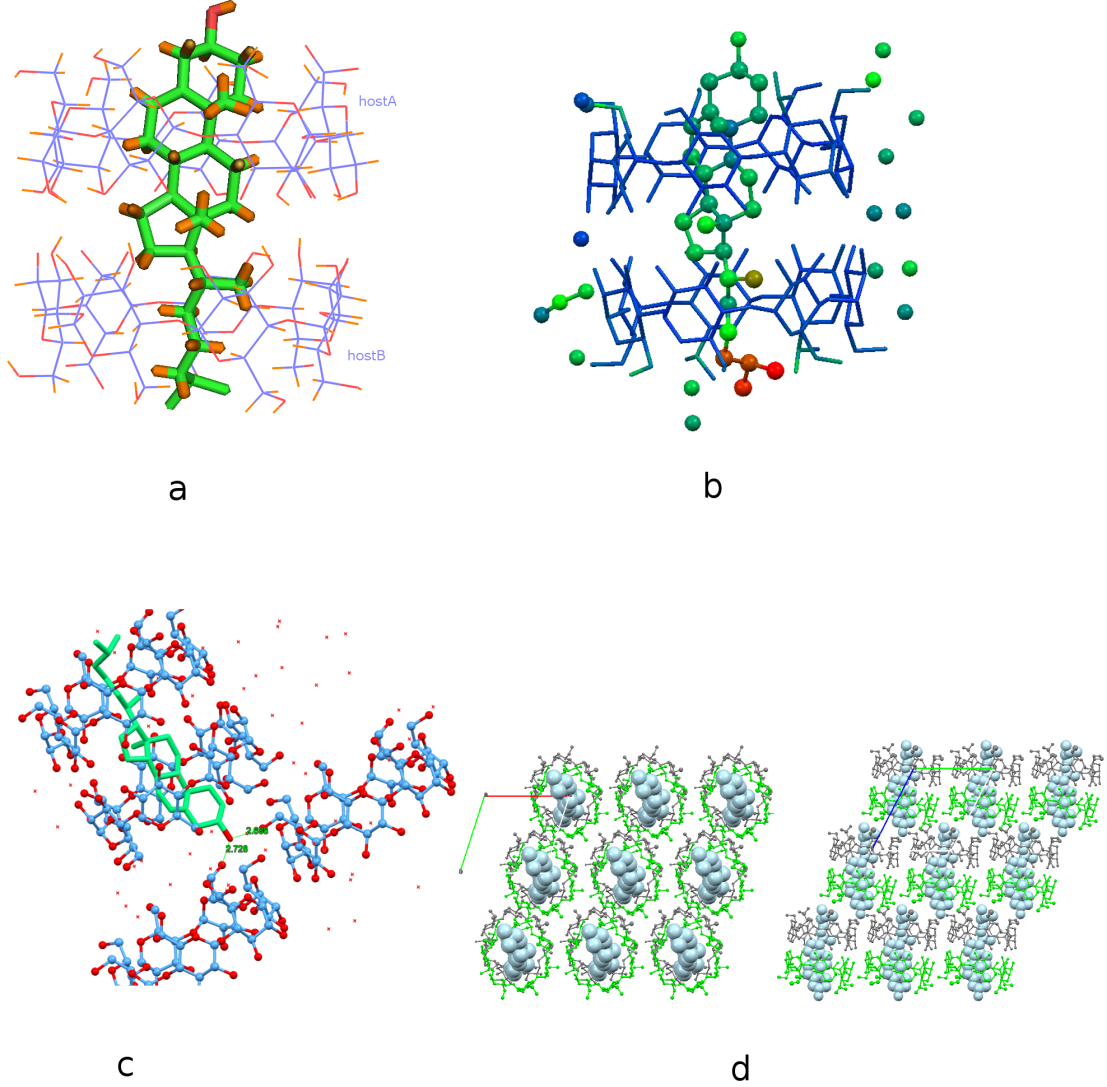
(a) Crystal structure of the inclusion compound of cholesterol in β-CD dimer. Water molecules are omitted for clarity. (b) The inclusion complex colored by atomic displacement parameters (*U*’s) using Mercury 3.9. The values of *U* increase from blue to red colour. (c) The hydroxy group of cholesterol is hydrogen bonded with the hydroxy groups of the primary rim of vicinal β-CD dimers. (d) Inclusion complexes stack along the crystallographic *c*-axis according to the Intermediate Channel (IM) packing mode. Projection along the *c*-axis (left) and *a*-axis (right).

The complex crystallizes in the *P*1 space group with lattice parameters quoted in Table 1. Its asymmetric unit contains two β-CD host molecules (denoted as host A and host B) arranged co-axially so that the secondary rim (head) of the one faces the secondary rim of the other forming a head-to-head dimer via intermolecular hydrogen bonds between their O3*n*-H hydroxy groups. A cholesterol molecule is found fully encapsulated inside the β-CD dimeric cavity, therefore the host:guest stoichiometry of the inclusion complex is 2:1 (Figure 2a). The unit cell contains also 22 water molecules distributed over 35 sites.

**Table 1:** Experimental details for the cholesterol/β-CD inclusion compound.

	cholesterol/β-CD

crystal data	

chemical formula	C_42_H_70_O_35_·C_42_H_70_O_35_·C_27_H_46_O·22(H_2_O)
*M*_r_	1504.94
crystal system, space group	triclinic, *P*1
temperature (K)	100
*a*, *b*, *c* (Å)	15.16 (3), 15.60 (3), 17.84 (3)
α, β, γ (°)	114.02 (14), 99.33 (13), 102.08 (12)
*V* (Å^3^)	3623 (12)
radiation type	Cu Kα
m (mm^-1^)	1.06
crystal size (mm)	0.23 × 0.12 × 0.07

data collection	

diffractometer	Bruker *APEX*-II
absorption correction	multi-scan *SADABS2016*/2 - Bruker AXS area detector scaling and absorption correction
*T*_min_, *T*_max_	0.498, 0.75
no. of measured, independent and observed [*I* > 2s(*I*)] reflections	61231, 15060, 11737
*R*_int_	0.105
θ_max_ (°)	50.9
(sin θ/λ)_max_ (Å^-1^)	0.504

refinement	

*R*[*F*^2^ > 2s(*F*^2^)], *wR*(*F*^2^), *S*	0.083, 0.224, 1.03
no. of reflections	15060
no. of parameters	1153
no. of restraints	82
H-atom treatment	H-atom parameters constrained
Dρ_max_, Dρ_min_ (e Å^−3^)	0.71, -0.43
absolute structure	Flack x determined using 4403 quotients [(I+)-(I-)]/[(I+)+(I-)] (Parsons, Flack and Wagner, Acta Cryst. B **2013,** 69, 249-259).
absolute structure parameter	0.04 (13)

The cholesterol molecule is accommodated ‘axially’ in the β-CD dimeric cavity. The mean plane of its ABCD ring system is perpendicular to the mean plane of the glucosidic O4*n* atoms of the hosts forming an angle of 83.18 (16)° with it. The sterol group of the guest is tightly fitted inside the hydrophobic cavity of the host A whereas its aliphatic ‘tail’ is located inside the hydrophobic cavity of the other host (host B) and characterized by high atomic displacement parameters in an effort to fill the “free” space inside the host B cavity (Figure 2b). The hydroxy group of cholesterol protrudes from the primary rim of host A (distance between the oxygen atom of the CHL hydroxy group and the mean plane of O4n atoms of host A is 5.329 (12) Å), hydrogen bonded with primary hydroxy groups of vicinal β-CD dimers (O1···O63B(1+*x*, *y*, *z*) = 2.688(4) Å, and O1···O66B(*x*, −1+*y*, *z*) = 2.721(5) Å,) aiding the crystal packing and also affecting the inclusion depth of the guest in the crystalline state (Figure 2c). The inclusion complex gains stability from numerous van der Waals and C–H···O interactions mainly between the guest and the inner dimeric host cavity. The observed host–guest interactions along with the extended hydrogen bond network between water molecules, hosts and guest are listed analytically in Supporting Information File 1, Table S1.

In particular, the side of C1 and C2 atoms of ring A and the opposite side of C6 and C7 atoms of ring B of CHL form closed-shell weak H···H interactions with the inner hydrogens (H5 and H3) of the oppositely located glucopyranoses of host A (G2, G3 and G6, G7, respectively). C2–H2A of ring A and C7–H7A of ring B also form C–H···O bonds with the O63C primary hydroxy group (partially occupied site, sof = 0.4) and the glycosidic O46A atom, respectively. The tight fit of the sterol group of the guest in host A hydrophobic cavity, is further enhanced by the H···H interactions between the hydrogens of the C19 methyl group, which is perpendicular to the mean plane of the sterol’s ring system, and the inner H5 hydrogens of the 3rd and 4th glucopyranose of host A macrocycle. The C18 methyl group of the guest, which has the same orientation with that of C19, is located at the interface of the β-CD dimer and does not interact with the host molecules. The C21 cholesterol methyl group being perpendicular to the C18 and C19 methyl groups is located in the host B macrocycle cavity and is in close contact with the inner H3 atom of its 6th glucopyranose unit (H36C–C36B). The aliphatic tail of the cholesterol molecule protrudes from the primary rim of host B. The hydrogens of its secondary C24 atom form C–H···O bonds with partially occupied water molecule sites located in the interdimeric space. The isopropyl group of the guest projects through the primary hydroxy rim of host B, clearly higher disordered than the sterol group, forming C–H···O bonds with the partially occupied primary hydroxy group O61D (sof = 0.2) of host B, disordered water molecules located in the dimers’ interspace and the primary hydroxy group O66A of host A of the adjacent dimer (1+*x*, *y*, -1+*z*).

Supporting Information File 1, Table S2 lists some parameters defining the conformation of the host molecules. The glucosidic O4*n* atoms in both host molecules form nearly regular heptagons, which are essentially planar, as indicated by their distances from their approximate centroids (*D*_K_), the distance between adjacent O4*n* atoms (*D*) and their deviations (*d*) from the O4*n* mean plane. The glycosidic residues have positive tilt angles, indicating that their primary sides incline towards the approximate sevenfold axis of the cavity. The majority of hydroxy groups in both host A and host B have the *gauche–gauche* conformation pointing outwards the cavity. One disordered hydroxy group in host A and two in host B illustrate both *gauche–gauche* and *gauche–trans* conformations pointing inwards and outwards the cavity.

The β-CD dimers stack along *c*-axis, the angle between their approximate seven-fold axis and *c*-axis being 7.86°, and form layers along the *a–b* crystal plane. The shift between two successive dimers along the *c*-axis is 5.91 Å. This displacement is very close to the average of 6 Å observed in the cases of the dimeric structures crystallizing according to the intermediate channel (IM) packing mode [30]. Therefore, the packing mode of the dimeric structure is characterized as IM (Figure 2d).

According to the classification of dimeric β-CD inclusion complexes [31], the dimers crystallizing in the *P*1 space group stack either according to the channel (CH) packing mode, if their cell dimensions are all about 15.5 Å, or to the IM packing mode in the case that one of the cell dimensions is more than 17 Å the two others being also about 15.5 Å. The Cambridge Structural Database (CSD) [32] search resulted in 26 structures of inclusion compounds in β-CD with similar cell dimensions. Among them, three entries (ANAXAP [33], UJEFEV [34] and XAMDEX [35]) are found to have a 2:1 host/guest stoichiometry. In all these cases, the shift between two successive dimers along the *c*-axis is 6.017, 6.22 and 6.27 Å, respectively. Thus they crystallize also in the IM packing mode.

The first case (ANAXAP), concerns the inclusion of a small molecule model of the [FeFe]-H_2_ase active site, (μ-SCH_2_NH(C_6_H_4_SO_3_^−^)CH_2_S)[Fe^I^(CO)_3_]_2_, within the cavity of a 1·2 β-CD sodium salt clathrate. 28 water molecules are also found in the asymmetric unit. The incorporation of charged functional groups into the guest molecule of cyclodextrin host/guest system provides a degree of stability to the inclusion complex. The head–head β-CD dimers formed by units interacting through hydrogen bonds between their secondary hydroxy groups is further stabilized through ion–dipole interactions with a Na^+^ counterion. This Na^+^ links the two β-CD’s together and also interacts with neighboring units in the extended two-dimensional crystalline array.

In the 8-hydroxyquinoline inclusion complex crystal structure (UJEFEV), the asymmetric unit consists of two β-CDs, one 8-hydroxyquinoline, two ethanol and thirty water molecules. The hydrophobic cavities of the two β-CDs forming a head-to-head dimer, contain only ethanol molecules whereas the small, planar 8-hydroxyquinoline molecule is found not being encapsulated but entrapped in a sandwich mode in the interface of the β-CD dimer.

The crystal structure of phenoxodiol/β-CD inclusion complex (XAMDEX) is the only case similar to the CHL/β-CD inclusion complex. Phenoxodiol is an isoflavone analogue that possesses potent anticancer properties. The asymmetric unit of phenoxodiol/β-CD includes one guest molecule encapsulated by two β-CD molecules, and twenty-six water molecules. All the water molecules surround the external area of the complex bridging the adjacent dimers. The guest phenoxodiol having a length of about 12 Å matches well with the length of the double barrel unit of two β-CDs (≈14.5 Å) and its terminal hydroxy groups make O–H···O hydrogen bonding contacts with water molecules. However, only one C–H···O bond between the oxygen atom of the guest’s benzopyran and an internal hydrogen of the host (C33–H33) is observed in the crystalline state. Thus, the guest is held in the dimeric β-CD cavity mainly via bridging molecules of water. Such formation of the inclusion complex is favourable for the facile release of the guest. Indeed, the phenoxodiol molecule is found having an occupancy factor of 0.5 in the β-CD dimeric cavity assumed able to be diffused through the crystal channels with the aid of the water network and the structure is characterized as ‘ship-in-a-bottle’.

On the other hand, in the case of the CHL/β-CD complex, the bulky cholesterol molecule with a length of about 16.5 Å is encapsulated with its hydroxy group and isopropyl terminal groups protruding from the primary hydroxy rim of the hosts and directly bonded with the hosts of the adjacent complex units, its tight fit in the β-CD dimeric cavity further supported by the above mentioned guest–host interactions. Therefore, cholesterol is always found in the cavity of the β-CD dimer, its size and shape prohibiting its diffusion in the crystal.

### Molecular dynamics

**Figure 3:**
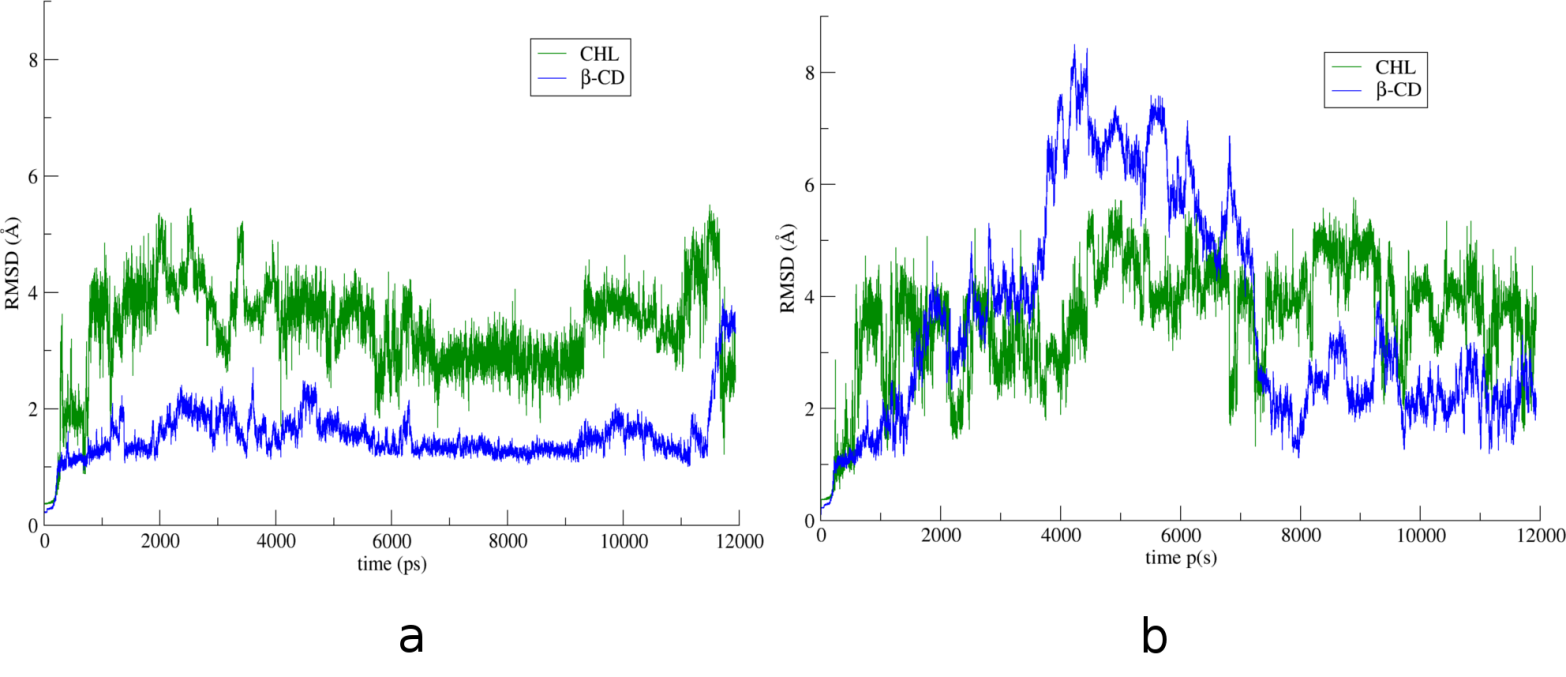
RMSD over time for all CHL (green) and β-CD (blue) atoms (a) at 300 K and (b) at 340 K.

**Figure 4:**
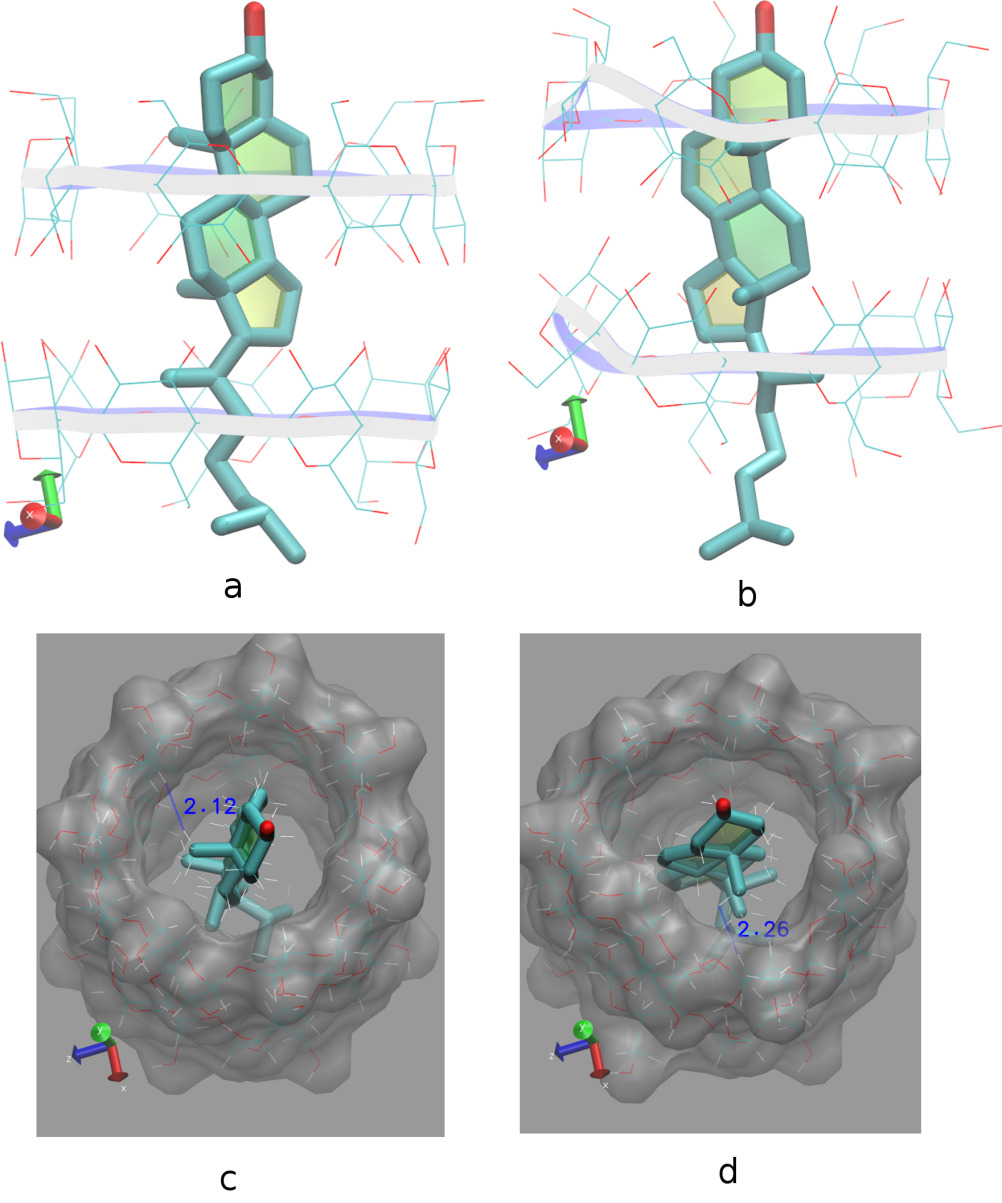
Representative snapshots of the CHL/β-CD inclusion complex at 0 (a, c) and 11 ns (b, d) in timescale and at 300 K. Water molecules are omitted for clarity. (a, b) Shift of the sterol group of the CHL molecule towards the β-CD dimeric interface. (c, d) H–H interactions between CHL ring A and H5 (initially) or H3 (subsequently) of host A are retained although CHL rotates about hosts’ seven-fold molecular axes. Image rendering was obtained with the VMD visualization program [36].

**Figure 5:**
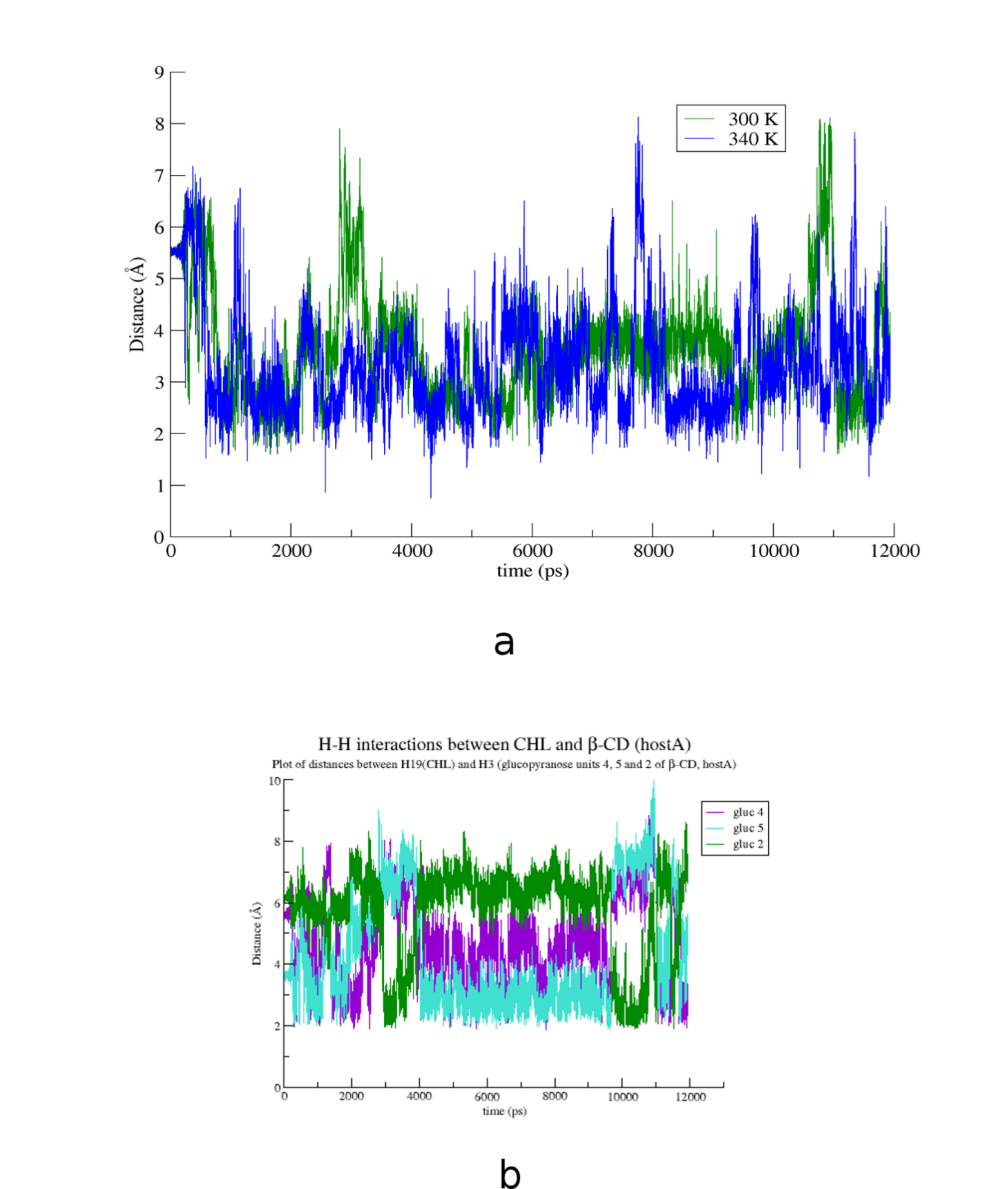
(a) Distance between the O1 atom (CHL) and the centroid *D*_K_ of the O4*n* atoms of host A at 300 (green) and 340 K (blue) and (b) distances between Η19 atom of CHL (ring A) and H3 atoms of the 4th (purple), 5th (cyan) and 2nd (green) glucopyranose units of host A.

The crystallographically determined atomic coordinates of the CHL/β-CD complex (host/guest stoichiometry 2:1) (Figure 2a) were subjected to equilibration and subsequent molecular dynamics simulations at both 300 and 340 K in explicit water solvent for almost 12 ns with the aim to monitor the dynamic behavior of CHL in β-CD in two different temperatures, study the host–guest interactions during the simulation time frame and calculate the host–guest binding affinity in each case. By monitoring the frames during the time interval of the simulations, we observed that the sterol group of the guest cholesterol molecule remains encapsulated inside the hydrophobic β-CD dimeric cavity while its aliphatic tail protrudes from the primary rim of the host B to the solvent in both cases. Figure 3 shows the time evolution of the root mean square deviation (RMSD) from the initial structure, calculated for all CHL (green) and β-CD (blue) atoms in their complex at 300 K (a) and 340 K (b). RMSD for CHL and β-CD are higher in the latter case. Although a significant deformation of the β-CD dimer is observed at 340 K, the dimer is not decomposed during the time frame of this simulation. In both examined cases the CHL molecule shifts from its original position towards the interface of the dimer (Figure 4a and b). This shift is favored by the lack of the crystal contacts between the hydroxy group of the guest and vicinal inclusion complexes occurring in the crystalline state (Figure 2c). In Figure 5a, the plot of the distance between the O1 atom of the guest and the centroid of the O4*n* atoms of host A during the simulation is given showing this shift and thus a preference of the sterol ring to be accommodated closer to the dimeric interface compared to its initial crystallographically observed site. Moreover, hydrogen–hydrogen interactions that occur initially between ring A atoms (CHL) and H5 (host A), after minimization and MD run of the system are observed between ring A atoms (CHL) and H3 (host A) (Figure 4c and d). Although the CHL guest molecule rotates about the seven-fold host molecule axis, the interactions between the hydrogen atoms of its sterol rings A and B with those of the wide rim of β-CD (H3) observed also by ^1^H NMR [26] are retained during the whole 12 ns simulation (Figure 5b).

The binding affinity of the CHL/β-CD complexes in aqueous solution has been previously calculated for different orientations of CHL in β-CD monomers as well as CHL encapsulation in head–head, head–tail and tail–tail dimers [24–25,33]. In this study, the determination by X-ray crystallography of the CHL inclusion in a head–head β-CD dimer conclusively resolves this ambiguity. The binding affinity calculations based on this model give high absolute Δ*G* values at both 300 K and 340 K temperatures (Table 2) indicating a very stable inclusion complex even at high temperatures. Van der Waals intermolecular forces are the predominant interactions sustaining the complex stability in aqueous solution (Table 2).

**Table 2:** Binding free energies (kcal/mole) resulting from MM/GBSA (and normal mode for the entropic term) analysis of the cholesterol/β-CD inclusion compound.

energy component	average energy (std. dev.)	

	*T* = 300 K	*T* = 340 K
	
Δ*E*_vdW_	−59.4 (±2.5)	−58.2 ( ±2.6)
Δ*E*_ele_	−1.9 (±2.4)	−2.0 (±2.3)
Δ*E*_GB_	24.5 (±3.4)	24.6 (±3.5)
Δ*E*_surf_	−5.9 (±0.2)	−5.9 (±0.3)
Δ*G*_gas_	−61.1 (±3.4)	−60.1 (±3.7)
Δ*G*_solv_	18.6 (±3.3)	18.8 (±3.3)
*Δ*G*_GB_	−42.5 (±2.6)	−41.3 (±2.8)
T∙Δ*S*	−23.0 (±3.6)	−22.00 (±3.9)
**Δ*G*_bind_	−19.5 (±4.4)	−19.3 (±4.8)

Δ*E*_vdW_ = van der Waals contribution from MM; Δ*E*_ele_ = electrostatic energy as calculated by the MM force field; Δ*E*_GB_ = the electrostatic contribution to the solvation free energy, calculated by GB model; Δ*E*_surf_ = nonpolar contribution to the solvation free energy, calculated by an empirical model; *Δ*G*_GB_ = Δ*G*_solv_ + Δ*G*_gas_; **Δ*G*_bind_ = Δ*G*_GB_ − T∙Δ*S*.

## Conclusion

The crystal structure of CHL in β-CD reveals the formation of a 2:1 host:guest inclusion complex. CHL is found encapsulated axially in a head-to-head β-CD dimer (host A and host B), tightly bound via numerous van der Waals and C–H···O interactions to the inner dimeric host cavity. The hydroxy group and the isopropyl group of the guest protrude from the primary rim of the host A and host B, respectively, forming crystal contacts with vicinal dimers and water molecules. The CHL/β-CD inclusion complex crystallizes in the triclinic space group *P*1 and the dimers are stacked along the *c*-axis according to the IM Channel packing mode.

In previous studies, the proximity between ring A and ring B protons of cholesterol and the secondary rim protons of β-CD were indicated by NMR concluding to a probable 2:1 host/guest inclusion formation [26]; the formation of 1:1 stoichiometric complexes pointed out by phase solubility [25] or MD studies [29]; favorable inclusion complex formation with β-CD dimer was shown by molecular modeling, proposing a tail–tail dimer [37] and finally MD calculations concluded that efficient removal of cholesterol from membranes requires the presence of β-CD dimers [27]. In this work, the crystallographic analysis of CHL/β-CD complex conclusively clarifies the inclusion mode of CHL in β-CD head–head dimers.

The MD simulations that performed based on the crystallographically determined model show that the inclusion complex is very stable in aqueous solution at both 300 and 340 K. In the absence of crystal contacts, van der Waals intermolecular forces are the predominant interactions sustaining the complex stability in aqueous solution. The interactions between the hydrogen atoms of the sterol rings A and B of cholesterol and those of the wide rim of β-CD which have been also reported previously by ^1^H NMR studies [26] are retained during the time frame of the MD simulations. Moreover, the high binding affinity values Δ*G*, estimated at 300 K and 340 K (−19.5 and −19.3 kcal/mol, respectively), indicate that the encapsulation of cholesterol in β-CD head–head dimer vastly increases the affinity of the CHL/β-CD inclusion complex compared to models of 1:1 host/guest stoichiometry (−3.3 kcal/mol, [25]) forming a very stable inclusion complex even at high temperatures.

## Experimental

### Chemicals

Cholesterol (*M*_w_ = 386.65 g/mol, 99% purity) and β-CD (*M*_w_ = 1135, 99% purity) were purchased from Fluka.

### Sample preparation

Crystals of the inclusion compound of CHL/β-CD were formed by using the slow cooling crystallization technique. More specific, 10.22 mg (0.026 mmoles) of CHL were added in a 2 mL equimolar aqueous solution of β-CD. The mixture was stirred at 70 °C for 4 h and gradually cooled to room temperature over a period of seven days.

### X-ray crystallography

A prismatic colorless specimen was used for single crystal X-ray diffraction data collection. The X-ray intensity data were measured at 100(2) K on a Bruker D8-VENTURE diffractometer, using a Cu Kα radiation (λ = 1.54178 Å) and an Oxford Cryosystems low-temperature device. A total of 3092 frames were collected during the 23.77 hour of total exposure time. Data integration using a narrow-frame algorithm and global-cell refinement were performed with the Bruker SAINT software package [38]. Data were corrected for absorption effects using the Multi-Scan method (SADABS) [38]. The ratio of minimum to maximum apparent transmission was 0.664.

The structure has been solved by intrinsic phasing method with SHELXT [39] and refined by full-matrix least squares against *F*^2^ using SHELXL-2014/7 [40] through the SHELXLE GUI [41] giving a final *R*_1_ index of ≈0.10. Due to the structural complexity of the inclusion compound, soft restraints on bond lengths and angles of the host and guest molecules were applied using the PRODRG2 webserver [42] and along with DFIX, DANG and FLAT commands in SHELXL. H-atoms of β-CD and cholesterol molecules were placed geometrically for temperature of 100 K and allowed to ride on the parent atoms by using SHELXL. H-atoms belonging to the disorder water molecules were not placed during refinement. *U*_iso_(H) values were assigned in the range 1.2–1.5 times *U*_eq_ of the parent atom. In order to maintain a high (>6.7) data/parameters ratio, anisotropic thermal parameters were imposed only to O2, O3 and O6 atoms of the host molecules. The graphic programs used to illustrate the crystal structures are Mercury 3.9 [43] and Olex2 [44]. Selected details of structure refinement along with important statistics are given in Table 1. The data can be obtained from The Cambridge Crystallographic Data Centre under the reference number CCDC 1571522.

### Molecular dynamics

MD simulation in explicit solvent environment using the crystallographically determined atomic coordinates of the β-CD dimer and the cholesterol guest molecule as the starting system was carried out. The Amber12 program [45] was used for all calculations and data analysis. The CLYCAM_06 force field [46] was applied to the atoms of β-CD, whereas GAFF parameters and AM1BCC charges were applied to the guest molecule using ANTECHAMBER [47]. The explicit solvent model TIP3P was used for water forming a periodic, octahedral box of at least 10 Å between the box walls and the complex. The formation of the thick water shell around the structures and the addition of hydrogen atoms in the two systems were performed with xLEaP. The program SANDER was used for both minimization and MD runs. The particle mesh Ewald method with the nonbonded cutoff distance set to 10 Å was used to create periodic boundary conditions. Temperature and pressure controls were performed using a Berendsen-type algorithm with coupling constants of 0.5 ps (equilibration) or 1.0 ps (production). The following protocol was applied: (a) energy minimization for hydrogen atoms, (b) 50 ps equilibration of the water molecules in the canonical ensemble (NVT) using 50 kcal mol^−1^ Å^−2^ positional restraints on the complex atoms, (c) unrestrained energy minimization of the system, (d) gradual temperature increase from 5 to 300 K or 340 K with 10 kcal mol^−1^ Å^−2^ restraints on the complex atoms, (e) gradual release of the restraints at 300 K or 340 K, (f) density equilibration in the isobaric–isothermal ensemble (NPT) for 250 ps and (g) MD run for 400 ps at 1 atm and 300 K or 340 K in the NPT ensemble. Production runs were carried out at 1 atm and 300 K or 340 K conditions for an additional time of 11 ns in the NPT ensemble. The trajectories were analyzed in order (a) to calculate the RMSD for both host and guest molecules and (b) monitor the values of some important geometric features during the MD calculations using CPPTRAJ [48]. The figure illustration, the video preparation and some geometric calculations of the MDs were performed using the program VMD 1.9.2 [36]. Moreover, the molecular mechanics/generalized Born surface area (MM/GBSA) method [49], was used for a theoritical estimation of the binding free energy Δ*G*_GB_ of the inclusion complex. The calculations were performed using 10,000 complex frames. Generalized Born ESURF calculated using 'LCPO' surface areas. The Δ*G*_GB_ value includes the terms Δ*G*_gas_ (van der Waals contribution from MM and the electrostatic energy as calculated by the MM force field) and Δ*G*_solv_ (the electrostatic contribution to the solvation free energy calculated by GB model and nonpolar contribution to the solvation free energy calculated using 'LCPO' surface areas) Δ*G*_GB =_ Δ*G*_gas_ + Δ*G*_solv_ as described by Miller et al. [50]. The entropy term *T*∙Δ*S* was also calculated from normal mode analysis with constant temperature using the respective module of the Amber 12 suite and added to the Δ*G*_GB_ term according to: Δ*G*_bind_ = Δ*G*_GB_ − *T*∙Δ*S.*

The entropy term was calculated by taking snapshots every 100 frames for as long as the equilibrated system of the inclusion complex was subjected to MD simulations. However, it should be noted that the estimation of the entropy term is often problematic as the normal mode lacks information of the conformational entropy and alternative methods do not give converged results [51]. Thus, this term is usually omitted and the comparison between similar complexes is based on the Δ*G*_GB_ solely.

## Supporting Information

File 1 Geometrical characteristics of the crystal structure of the CHL/β-CD inclusion compound.
