# Supplementary material for: Crystal structure of the inclusion complex of cholesterol in β-cyclodextrin and molecular dynamics studies
Source: Beilstein J Org Chem. 2018 Apr 11;14:838–48. doi: 10.3762/bjoc.14.69 (PMC5905284; doi:10.3762/bjoc.14.69)
Supplement: File 1 — Geometrical characteristics of the crystal structure of the CHL/β-CD inclusion compound. [file Beilstein_J_Org_Chem-14-838-s001.pdf]

Supporting Information

for

Crystal structure of the inclusion complex of cholesterol in β-cyclodextrin and molecular dynamics studies

Elias Christoforides, Andreas Papaioannou and Kostas Bethanis*

Address: [1]Department of Biotechnology, Agricultural University of Athens, 75 Iera Odos, Athens 11855, Greece

Email: Kostas Bethanis - kbeth@aua.gr

*Corresponding author

Geometrical characteristics of the crystal structure of the CHL/βCD inclusion compound.

Table S1: a. Hydrogen bonds network of the crystal structure

		Distance (Å)	Symmetry operation
	*Between water molecules**		
1	OW1···OW2	2.782(15)	(x, y, z)
2	OW1···OW35	2.56(5)	(x,-1+y,z)
3	OW2···OW26	2.58(3)	(x, y, z)
4	OW3···OW14	2.786(18)	(x, y, z)
5	OW4···OW6	2.784(17)	(x, y, z)
6	OW7···OW11	2.898(18)	(x, y, z)
7	OW7···OW18	2.86(2)	(x, y, z)
8	OW7···OW32	2.74(3)	(x, y, z)
9	OW8···OW13	2.789(17)	(x, y, z)
10	OW9···OW16	2.76(2)	(x,y,1+z)
11	OW9···OW19	2.86(2)	(x, y, z)
12	OW9···OW33	2.71(8)	(x, y, z)
13	OW11···OW14	2.846(18)	(x, y, z)
14	OW14···OW19	2.78(2)	(x, y, z)
15	OW16···OW29	2.59(7)	(x, y, z)
16	OW1···OW15	2.849(18)	(x, -1+y, z)
17	OW2···OW10	2.747(19)	(x, y, z)
	*Between water molecules and the two hosts**		
1	OW6···O24A	2.645(15)	(-1+x, -1+y, z)
2	OW13···O63A	2.69(2)	(-1+x, -1+y, -1+z)
3	OW13···O61B	2.669(16)	(x, y, z)
4	OW16···O62B	2.75(3)	(x, y, z)
5	OW1···O67B	2.707(15)	(x, -1+y, z)
6	OW10···O23B	2.776(17)	(x, y, z)
7	OW12···O25A	2.738(16)	(-1+x, y, z)
8	OW12···O37A	2.884(15)	(x, y, z)
9	OW31···O32B	2.92(6)	(x, y, z)
10	OW34···O32B	2.69(13)	(x, y, z)
11	OW36···O22B	2.68(7)	(x, y, z)
	Between the two hosts		
1	O54A···O62D	2.81(2)	(1+x,1+y,1+z)
2	O63A···O52B	2.794(19)	(1+x,1+y,1+z)
3	O63A···O62D	2.89(2)	(1+x,1+y,1+z)

b. Host–guest intermolecular interactions

		Distance (Å)	Type of interaction **
Ring A of CHL	***hostA***		
C1-H1A	H52A-C52A	2.119(4)	1
C1-H1B	H52A-C53A	2.200(3)	1
C1-H1B	H53A-C53A	2.152(4)	1
C2-H2A	O63C (sof = 0.4)	2.50(3)	2
Methyl C19 of CHL	***hostA***		
C19-H19C	H53A-C53A	2.295(5)	1
C19-H19C	H54A-C54A	2.247(4)	1
C19-H19A	H54A-C54A	2.394(5)	1
Ring B of CHL	***hostA***		
C6-H6	H57A-C57A	2.313(5)	1
C6-H6	H56A-C56A	2.267(4)	1
C7-H7A	H37A-C37A	2.362(6)	1
C7-H7B	H36A-C36A	2.225(5)	1
C7-H7B	O46A	2.596(10)	2
Methyl C21 of CHL	***hostB***		
C21-H21B	H36C-C36B	2.376(4)	1
Aliphatic chain of CHL			
C24-H24E	OW29 (sof = 0.2)	1.99(7)	2
C24-H24B	OW29 (sof = 0.2)	2.61(7)	2
Isopropyl group of CHL*			
C26-H26E	O61D (hostB; sof = 0.2)	2.027(4)	2
C26-H26E	OW33 (x, -1+y, -1+z; sof = 0.2)	1.781(4)	2
C26-H26F	O66A (hostA; 1+x, y, -1+z)	2.648(5)	2
C26-H26G	OW29 (x, -1+y, -1+z; sof = 0.2)	2.041(3)	2
C26-H26G	O62B (x, -1+y, -1+z; sof = 0.4)	2.702(5)	2
C27-H27D	O61D (hostB; sof = 0.2)	2.452(5)	2

(*) H-atoms belonging to the disorder water molecules were not placed during refinement.

(**) Type 1: H···H closed shell interaction; Type 2: C–H···O interaction

Table S2: Conformation characteristics of the host molecules of cholesterol/β-CD inclusion compound.

	D_K(Å)	*D* (Å)	Φ (°)	*d* (Å)	τ (°)	*t* (°)	*C*
**hostA**							
n = 1	4.932(7)	4.343(9)	130.08(11)	-0.01129(3)	+6.616(19)	-63.42(13)	*gg*
n = 2	5.121(11)	4.369(7)	127.31(12`)	-0.006483(15)	+2.491(7)	-68.64(10)	*gg*
n = 3	5.147(11)	4.282(8)	126.44(11)	0.004086(9)	+8.62(3)	-63.40(14)<br>77.21(14)	*gg*<br>*gt*
n = 4	4.908(8)	4.546(10)	130.91(11)	0.002920(7)	+3.106(9)	-67.08(14)	*gg*
n = 5	4.962(9)	4.267(8)	131.01(11)	0.006961(16)	+12.29(3)	-64.10(16)	*gg*
n = 6	5.261(11)	4.340(7)	123.13(12)	-0.02692(6)	+8.46(3)	-66.02(17)	*gg*
n = 7	4.966(9)	4.49(1)	131.09(11)	0.03072(7)	+7.96(2)	-62.40(12)	*gg*
**hostB**							
n = 1	4.930(9)	4.374(8)	130.95(12)	0.002143(5)	+5.420(15)	-54.32(9)<br>81.04(11)	*gg*<br>*gt*
n = 2	5.257(11)	4.229(7)	123.02(11)	0.01677(4)	+8.60(3)	47.69(9)<br>-63.27(13)	*gt*<br>*gg*
n = 3	4.983(9)	4.465(9)	130.29(11)	-0.01739(4)	+6.50(2)	-68.81(13)	*gg*
n = 4	4.834(8)	4.343(9)	132.62(11)	0.000947(2)	+12.05(3)	-67.03(16)	*gg*
n = 5	5.145(10)	4.380(7)	125.50(12)	0.006105(14)	+9.92(3)	-66.65(17)	*gg*
n = 6	5.154(11)	4.276(8)	126.47(12)	0.005668(13)	+10.66(3)	-65.12(13)	*gg*
n = 7	4.898(8)	4.492(10)	131.14(10)	-0.01424(3)	+6.323(19)	-64.31(14)	*gg*

D_K(Å) = KA···O4*n*A; *D*(Å) = O4*n*···O4(*n*+1); Φ(°) = O4(*n*-1) ···O4*n*···O4(*n*+1) angles; *d*(Å) = deviations of the O4n atoms from their least squares plane ; τ(°) = tilt angles between the optimum O4*n* plane and the mean planes of the O4(*n*-1), C1*n*, C4*n* and O4*n* atoms; *t*(°) = O5*n*-C5*n*-C6*n*-O6*n* torsion angles; *C* = conformation of the primary hydroxyl groups.
